# Evaluation of GC/MS-Based ^13^C-Positional Approaches for TMS Derivatives of Organic and Amino Acids and Application to Plant ^13^C-Labeled Experiments

**DOI:** 10.3390/metabo13040466

**Published:** 2023-03-23

**Authors:** Younès Dellero, Olivier Filangi, Alain Bouchereau

**Affiliations:** 1 Institute for Genetics, Environment and Plant Protection (IGEPP), National Research Institute for Agriculture, Food and Environment (INRAE), Institut Agro, Université Rennes, 35650 Le Rheu, France; 2 Metabolic Profiling and Metabolomic Platform (P2M2), Biopolymers Interactions Assemblies, Institute for Genetics, Environment and Plant Protection, 35650 Le Rheu, France; 3 MetaboHUB, National Infrastructure of Metabolomics and Fluxomics, 35650 Le Rheu, France

**Keywords:** mass spectrometry, gas chromatography, isotope, brassica napus, photorespiration, tricarboxylic acid cycle, phosphoenolpyruvate carboxylase, glycine, serine

## Abstract

Analysis of plant metabolite ^13^C-enrichments with gas-chromatography mass spectrometry (GC/MS) has gained interest recently. By combining multiple fragments of a trimethylsilyl (TMS) derivative, ^13^C-positional enrichments can be calculated. However, this new approach may suffer from analytical biases depending on the fragments selected for calculation leading to significant errors in the final results. The goal of this study was to provide a framework for the validation of ^13^C-positional approaches and their application to plants based on some key metabolites (glycine, serine, glutamate, proline, α-alanine and malate). For this purpose, we used tailor-made ^13^C-PT standards, harboring known carbon isotopologue distributions and ^13^C-positional enrichments, to evaluate the reliability of GC-MS measurements and positional calculations. Overall, we showed that some mass fragments of proline_2TMS, glutamate_3TMS, malate_3TMS and α-alanine_2TMS had important biases for ^13^C measurements resulting in significant errors in the computational estimation of ^13^C-positional enrichments. Nevertheless, we validated a GC/MS-based ^13^C-positional approach for the following atomic positions: (i) C1 and C2 of glycine_3TMS, (ii) C1, C2 and C3 of serine_3TMS, and (iii) C1 of malate_3TMS and glutamate_3TMS. We successfully applied this approach to plant ^13^C-labeled experiments for investigating key metabolic fluxes of plant primary metabolism (photorespiration, tricarboxylic acid cycle and phosphoenolpyruvate carboxylase activity).

## 1. Introduction

Plant growth and seed yield establishment are supported at the molecular level by the activity of central metabolic pathways [1]. These pathways act together to sustain carbon and nutrient uptake, their assimilation and use and ultimately their remobilization from source-to-sink tissues [1,2,3]. Hence, it is essential to develop methods and tools to monitor the activity of these pathways. For this purpose, labeling experiments with stable isotopes (^13^C and ^15^N) was applied to plants and provided valuable information at different levels of integration [4]. Indeed, analysis of ^15^N-enrichments at the organ level after a pulse-chase experiment allowed the separate evaluation of the contribution of nitrogen uptake and remobilization processes to seed filling [5]. Conversely, labeling experiments with ^13^C or ^15^N metabolic probes followed by analysis of ^13^C or ^15^N enrichments at the metabolite level allowed the evaluation of protein turnover and carbon allocations to major metabolites, thereby reflecting the regulation of the activity of central metabolic pathways [6,7,8,9,10,11,12,13,14]. However, there may be more than one pathway contributing to the biosynthesis of some important metabolites [4,15]. Thus, access to positional information of ^13^C-enrichments is sometimes a prerequisite for determining the contributions of individual metabolic pathways [16]. This positional information may also help to resolve some flux identifiability issues encountered in metabolic flux analysis [17].

Analysis of ^13^C-enrichment in plant metabolites is generally performed using either Nuclear Magnetic Resonance (NMR) or Mass Spectrometry (MS). Although ^13^C-NMR analysis is less sensitive than MS analysis, it can directly give access to each carbon of a metabolite with a good resolution for separation in either purified or complex plant matrices [18]. Notably, ^13^CO_2_ incorporation into illuminated leaves combined with ^13^C-NMR analysis successfully determined the metabolic origin of carbon atoms in malate and glutamate by identifying the respective contributions of phosphoenolpyruvate carboxylase (PEPc) and the tricarboxylic acid (TCA) cycle [15,19,20]. ^13^C-NMR was also recently used to evaluate the concurrent contribution of mevalonate and methylerythritol-phosphate pathways to the biosynthesis of sclareol precursors [21]. Unlike ^13^C-NMR, analysis of ^13^C by MS is a very sensitive method, but it mainly provides information at the isotopologue level. Thus, the labeled molecules are separated according to their numbers of ^13^C atoms for a given metabolite (isotopologues) resulting in mass shifts from M0 non-labeled molecules [22]. Since an isotopologue can contain several isotopomers, i.e., molecules labeled with the same numbers of ^13^C but differing in position, the analysis of the results can be relatively more complex [23]. Nevertheless, this MS method was successfully applied to monitor the fate of ^13^C-metabolic probes within plant metabolism due to the ability to monitor some pathway-specific isotopologues [12,24,25,26,27,28]. Approaches using ^13^C-positional GC-EI-MS have emerged recently to improve the level of information obtained by MS [17,29,30]. These methods combined mean ^13^C-enrichments of multiple fragments from the same derivative to calculate ^13^C-positional enrichments. To date, this approach has been limited to the study of TMS derivatives for malate, glutamate and glucose in the field of plant science [29]. Indeed, the possibility to extrapolate this positional approach to multiple metabolites is restricted by the availability of the informative ions and by their intensity. A recent GC-MS study confirmed that the fragmentation pattern of TMS derivatives for succinate, citrate and fumarate prevented the use of this positional ^13^C approach [31]. Regarding TMS derivatives of amino acids, most of them showed only an intense ion resulting from an alpha-cleavage of the aliphatic carbon [32,33]. However, some amino acids, such as alpha-alanine, glycine, proline and serine, also have intense ions from additional fragmentation pathways (alpha-cleavage to nitrogen and loss of a methyl radical followed by CO elimination) and could be used to obtain valuable information on plant metabolism [32].

To date, ^13^C-positional approaches have been mainly applied to assess the contribution of PEPc activity to malate/glutamate biosynthesis and to gluconeogenesis in guard cells and leaves from Arabidopsis and tobacco [29,34]. However, this ^13^C-positional method did not consider potential errors of measurements associated to the MS fragments that could be derived from both analytical and matrixial biases [26,35,36]. In addition, these errors of measurements may easily be larger in the calculated ^13^C-positional enrichments by combining several biased fragments in several calculations. Finally, the proposed method used relative ^13^C-enrichment values normalized to the “0” time point of the experiment (division) before the calculations [29,34]. This normalization step is extremely dangerous because (i) no enrichment is expected at the “0” time point if measurements are highly accurate, except natural ^13^C abundance; and (ii) some mass fragments can have very poor accuracies at natural ^13^C abundance. These problems will distort the normalization scales between different fragments of a same TMS derivative, thereby affecting the accuracy of ^13^C-positional enrichment calculations. Thus, the reliability of the ^13^C-positional GC-MS approaches remain to be assessed prior to its wide application to plant ^13^C-labeled experiments. The accuracy of ^13^C measurements by GC-MS has been thoroughly evaluated recently at the isotopologue level using tailor-made ^13^C standards (^13^C-PT) harboring a fully controlled carbon isotopologue distribution (CID) pattern [26]. Interestingly, these standards had equal proportions of each possible isotopologue and each possible associated ^13^C isotopomer leading to a positional mean ^13^C-enrichment of 50% for each carbon of each metabolite [36]. Thus, these standards could be used to assess the accuracy of calculations from ^13^C-positional GC-MS approaches. Here, we took advantage of ^13^C-PT standard samples to evaluate such calculation accuracy for some important metabolites of plant metabolism (glycine, serine, glutamate, proline, α-alanine and malate). First, we selected multiple mass fragments from a same metabolite (TMS derivative) and assessed their CID patterns. Next, we used the mean ^13^C-enrichment of these fragments to calculate some ^13^C-positional enrichments. Finally, we applied the validated calculations to plant ^13^C-labeled experiments and reconnected some calculated ^13^C-positional enrichments to known metabolic processes regulated by light and dark conditions (photorespiration and PEPc/TCA cycle).

## 2. Materials and Methods

### 2.1. Positional Identification of Carbon Backbones from Mass Fragments of TMS Derivatives

For Glutamate_3TMS and Malate_3TMS, the identity of carbons contained in TMS derivative mass fragments was based on previously published studies that used metabolites labeled in specific positions [29,31]. For Proline_2TMS, we separately injected a non-labeled proline standard and a commercial U-^13^C-Proline standard (^13^C isotopic purity of 99%, Cambridge Isotope Laboratories (Eurisotop, Saint-Aubin, France)). For other TMS derivatives, we retrieved mass tables from GC-MS spectra of the Golm Metabolome Database corresponding to the injection of either ^13^C-reference substances or ^13^C-analytes produced with a determined ^13^C isotopic signature [37,38]. For each TMS derivative and each fragment analyzed, the mass isotopic cluster was used to calculate the area distribution for each *m*/*z* expressed as a percentage of the total area for the mass isotopic cluster (relative intensity from 0 to 100%).

### 2.2. Analysis of Carbon Isotopologue Distributions (CID) from ^13^C-PT Standards and Plant ^13^C-Labeled Experiments

We retrieved processed GC-MS spectra from two distinct GC-MS-based experiments. The first experiment consisted of the injection of tailor-made isotopic standards (^13^C-PT samples) [26]. These standards harbored a fully controlled carbon isotopologue distribution following a binomial pattern [36]. Consequently, all fragments produced from the TMS derivatives of these standards had a fractional mean ^13^C enrichment of 0.5 and their CIDs were predicted according to the number of carbons present within each mass fragment. The amino acids were analyzed with the ^13^C-PT protein hydrolysates (4 biological replicates), while malate was analyzed with the ^13^C-PT full metabolite extract (4 biological replicates). The second experiment consisted of the injection of 48 polar metabolite extracts from incorporation kinetics of U-^13^C-pyruvate into *Brassica napus* (*B. napus*) leaf discs in either light or dark conditions for up to 6 h (4 biological replicates per condition and per timepoint) [26]. The previous processing of the spectra for all samples comprised the adjustment of the retention time with a mixture of alkanes and the peak identification with respect to authentic standards derivatized and by reference to GC-EI-MS mass spectra of TMS derivatives in the US National Institute of Standards and Technology database and in the Golm metabolome database [38].

Then, the spectra were processed again with a modified method based on the new fragments presented in Table 1. Raw areas were subjected to correction for the contribution of naturally occurring isotopes of all elements except the carbon arising from the metabolite backbone using the IsoCor v2.1.4 package of Python (https://github.com/MetaSys-LISBP/IsoCor, accessed on 20 February 2023) in low-resolution mode. For samples arising from U-^13^C-pyruvate incorporations, an additional correction was performed to account for the contribution of naturally occurring isotopes of the carbon arising from the metabolite backbone and the isotopic purity of the tracer U-^13^C-pyruvate (99%). Practically, this correction led to a value of 0% if labeling was at natural ^13^C abundance (1.1%) and a value of 100% if labeling was at the maximal possible ^13^C enrichment. Samples of ^13^C-PT were not concerned by this additional correction since they already held fully controlled ^12^C/^13^C patterns at all positions. A corrective method dedicated to IsoCor was specifically created for the TMS-derivative fragments described in this study (Table 1). The IsoCor files and a GC-MS example file are available in the following INRAE dataverse, and all information will be updated indefinitely [39]. The method is publicly available and can also be run directly from GC-MS source files using a Galaxy Workflow URL available at the previously mentioned INRAE dataverse. For this purpose, each raw GC-MS dataset should contain a column “Name” for the fragments considered and a column “Area”. The name of each fragment must be written exactly as specified in the GC-MS example file from the previously mentioned INRAE dataverse. Fractional CIDs and fractional mean ^13^C enrichments were directly obtained from IsoCor outputs (isotopologue_fraction and mean_enrichment). For ^13^C-PT samples, CIDs were rescaled to Pascal’s Triangle coefficients by multiplying with 2^n^, where *n* is the number of carbon atoms [36].

### 2.3. Calculations of ^13^C-Positional Enrichments

^13^C-positional enrichments were calculated by combining mean ^13^C enrichments of several mass fragments that shared part of the carbon backbone from the same TMS derivative. A workflow has been created to calculate these ^13^C-positional enrichments directly from the outputs of our ^13^C-processing method with a URL and explanations available at the dedicated INRAE dataverse [39]. Briefly, by considering a metabolite X with 3 carbons, the mean ^13^C enrichment (M^13^C) of the mass fragment containing the C1-C2-C3 carbons (M^13^C_(X)_C1C2C3) can be written as a function of positional mean ^13^C enrichments (using fractional values here):

(1) $$M^{13}C_{\left(X\right)}C1C2C3=\frac{M^{13}C_{\left(X\right)}C1+M^{13}C_{\left(X\right)}C2+M^{13}C_{\left(X\right)}C3}{3}$$

Considering another fragment containing the C2-C3 carbons, the mean ^13^C enrichment (M^13^C(X)_C2-C3_) is calculated by:

(2) $$M^{13}C_{\left(X\right)}C2C3=\frac{M^{13}C_{\left(X\right)}C2+M^{13}C_{\left(X\right)}C3}{2}$$

Then, the mean ^13^C enrichment for the C1 carbon (M^13^C(X)_C1_) can be calculated by:

M^13^C_(X)_C1 = (3 × M^13^C_(X)_C1C2C3) − (2 × M^13^C_(X)_C2C3) (3)

Following the same rationale, we calculated some ^13^C-positional enrichments based on the mass fragments detailed in Table 1. For alanine_2TMS, we used the following equations by combining the C2C3 fragment at either *m*/*z* 116 or 190 with C1 and C1C2 fragments:

(4) $$M^{13}C_{\left(\mathrm{Alanine}\right)}C1C2C3=\frac{{M^{13}C_{\left(Alanine\right)}C1+M}^{13}C_{\left(Alanine\right)}C2C3}{2}\;M^{13}C_{\left(\mathrm{Alanine}\right)}C2=(2\times M^{13}C_{\left(\mathrm{Alanine}\right)}C1C2)-M^{13}C_{\left(\mathrm{Alanine}\right)}C1\;M^{13}C_{\left(\mathrm{Alanine}\right)}C3=(2\times M^{13}C_{\left(\mathrm{Alanine}\right)}C2C3)-M^{13}C_{\left(\mathrm{Alanine}\right)}C2$$

For glutamate_3TMS, we used the following equation by combining the C2C3C4C5 fragment at either *m*/*z* 156 or 246 with the C1C2C3C4C5 fragment:

M^13^C_(Glutamate)_C1 = (5 × M^13^C_(Glutamate)_C1C2C3C4C5) − (4 × M^13^C_(Glutamate)_C2C3C4C5) (5)

For glycine_3TMS, we used the following equation by combining the C2 fragment at either *m*/*z* 174, 100 or 86 with the C1C2 fragment:

M^13^C_(Glycine)_C1 = (2 × M^13^C_(Glycine)_C1C2) − M^13^C_(Glycine)_C2 (6)

For proline_2TMS, we used the following equation by combining the C2C3C4C5 fragment at either *m*/*z* 142 or 216 with the C1C2C3C4C5 fragment:

M^13^C_(Proline)_C1 = (5 × M^13^C_(Proline)_C1C2C3C4C5) − (4 × M^13^C_(Proline)_C2C3C4C5) (7)

For serine_3TMS, we used the following equations by combining the C1C2 and C2C3 (*m*/*z* 204) fragments with either the C2 fragment (Method A; Equation (8)) or the C1C2C3 fragment (Method B; Equation (9)):

M^13^C_(Serine)_C1 = (2 × M^13^C_(Serine)_C1C2) − M^13^C_(Serine)_C2 M^13^C_(Serine)_C3 = (2 × M^13^C_(Serine)_C2C3) − M^13^C_(Serine)_C2 M^13^C_(Serine)_C1C2C3 = M^13^C_(Serine)_C1 + M^13^C_(Serine)_C2 + M^13^C_(Serine)_C3 (8)

M^13^C_(Serine)_C1 = (3 × M^13^C_(Serine)_C1C2C3) − (2 × M^13^C_(Serine)_C1C2) M^13^C_(Serine)_C3 = (3 × M^13^C_(Serine)_C1C2C3) − (2 × M^13^C_(Serine)_C2C3) M^13^C_(Serine)_C1 = (3 × M^13^C_(Serine)_C1C2C3) − (M^13^C_(Serine)_C1 + M^13^C_(Serine)_C1C2) (9)

For malate_3TMS, we used the following equations by combining the C1C2C3C4 fragment at either *m/z* 335 or 245 with the C2, C2C3 and C2C3C4 fragments:

M^13^C_(Malate)_C1 = (4 × M^13^C_(Malate)_C1C2C3C4) − (3 × M^13^C_(Malate)_C2C3C4) M^13^C_(Malate)_C4 = (3 × M^13^C_(Malate)_C2C3C4) − (2 × M^13^C_(Malate)_C2C3) M^13^C_(Malate)_C3 = (3 × M^13^C_(Malate)_C2C3C4) − (M^13^C_(Malate)_C2 + M^13^C_(Malate)_C4) M^13^C_(Malate)_C3 = (4 × M^13^C_(Malate)_C1C2C3C4) − (M^13^C_(Malate)_C1 + M^13^C_(Malate)_C2 + M^13^C_(Malate)_C4) (10)

The C3 ^13^C-positional enrichment of malate_3TMS was also calculated by combining the C2, C4 and C2C3C4 fragments:

M^13^C_(Malate)_C3C4 = (3 × M^13^C_(Malate)_C2C3C4) − (M^13^C_(Malate)_C2) M^13^C_(Malate)_C3 = (2 × M^13^C_(Malate)_C3C4) − (M^13^C_(Malate)_C4) (11)

### 2.4. Data Management, Visualization and Statistical Analysis

Raw data were retreated and visualized using custom R scripts based on the packages tidyr, dplyr and ggplot2 [40,41,42]. For ^13^C-PT standards, the normal distribution of data was assumed based on the observed normal distribution for measurement errors (CID accuracy) [26,35]. Thus, comparison of measured/calculated values with theorical (expected) values was based on the 95% confidence intervals calculated with a Student’s t-test table: (mean − 1.96 * (SD/√(number of samples); mean + 1.96 * (SD/√(number of samples)). For plant ^13^C-labeled experiments, the data did not follow a normal distribution based on a Shapiro–Wilk test for each group of mean values (1 metabolite/1 fragment/1 time point/1 condition). Thus, statistical comparisons of two groups of mean values was achieved with a Wilcoxon–Mann–Whitney test (*p*-value < 0.05, two sided) using built-in functions from Rstudio v2022.07.2 build 576 [43].

## 3. Results

A recent study previously reported the use of some fragments for a ^13^C-positional GC-MS approach without a reliable evaluation of measurement accuracy [29]. Here, we evaluated this accuracy for both compounds (malate and glutamate) but also for some key metabolites of plant central metabolism (glycine, serine, proline and α-alanine). These last metabolites had promising fragmentation patterns for ^13^C-positional approaches (Table 1). Besides this, glycine and serine could also be used to follow the metabolic pathways involved in serine biosynthesis (glycolysis versus photorespiration), while α-alanine could be used as a proxy for pyruvate (same carbon backbone). Finally, proline could also be used as a proxy to evaluate PEPc contribution to glutamate biosynthesis.

### 3.1. Selection of Mass Fragments

**Figure 1:**
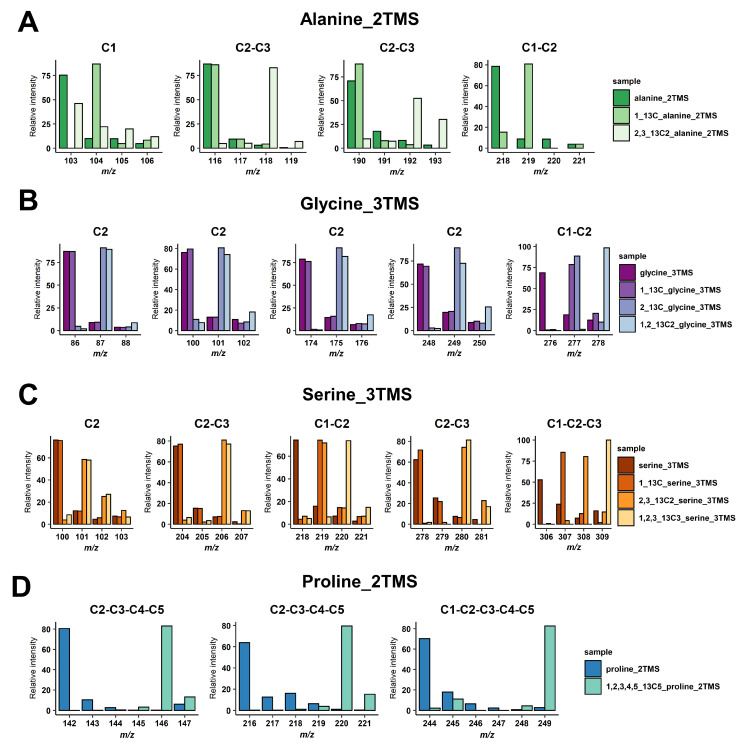
Identification of carbons contained in the mass fragments. (**A**) Alanine_2TMS, (**B**) glycine_3TMS, (**C**) serine_3TMS, and (**D**) proline_2TMS. The presence of ^13^C atoms from the original metabolite introduced mass shifts in the fragments of the TMS derivative analyzed.

For malate and glutamate, we took advantage of previously identified fragments for malate_3TMS and glutamate_3TMS derivatives [29,31], summarized in Table 1. These fragments allowed for the calculation of all ^13^C-positional enrichments for malate and C1 ^13^C-enrichment for glutamate (reflecting potentially PEPc contribution). For others, we performed injections of ^13^C-reference substances or analyzed GC-MS spectra of the Golm Metabolome Database corresponding to the injection of either ^13^C-reference substances or ^13^C analytes produced with a controlled ^13^C pattern [37,38]. The molecular losses and chemical formulas of mass fragments were then determined according to common rules of mass fragmentations for amino acids [32] (Table 1). Overall, the identity of the carbons contained in each mass fragment was confirmed by mass shifts with ^13^C-labeled molecules (Figure 1). For alanine_2TMS, we obtained mass fragments containing C1, C2-C3 and C1-C2 carbons allowing us to perform multiple calculations to get access to ^13^C enrichments at C2 and C3 positions (Figure 1A). For glycine_3TMS, there were several mass fragments containing the C2 carbon, thus allowing us to select the most accurate fragments for calculations of ^13^C enrichment at C1 position (Figure 1B). For serine_3TMS, we obtained fragments containing C2, C1-C2, C2-C3 and C1-C2-C3 carbons, thus allowing us to calculate ^13^C enrichments at C1 and C3 positions with two possible combinations of mass fragments (based on C2 or on C1-C2-C3 fragments) (Figure 1C). Finally, there were three mass fragments for proline (two C2-C3-C4-C5 and one C1-C2-C3-C4-C5) for calculating ^13^C enrichment at the C1 position and identifying a potential PEPc contribution.

### 3.2. Validation of GC/MS Based ^13^C-Positional Approaches

**Figure 2:**
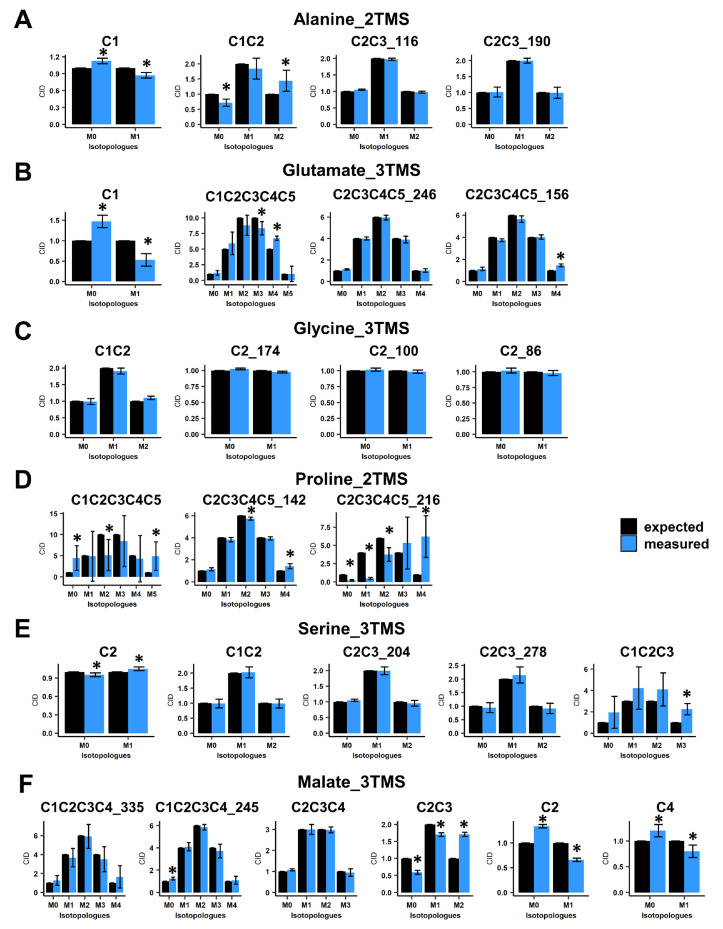
Evaluation of carbon isotopologue distribution measurements for the selected mass fragments using ^13^C-PT standards. (**A**) Alanine_2TMS, (**B**) glutamate_3TMS, (**C**) glycine_3TMS, (**D**) serine_3TMS, (**E**) proline_2TMS, and (**F**) malate_3TMS. Black bars, expected CID; blue bars, measured CID. Values represent the mean ± SD for four biological replicates. Statistical differences between predicted and measured CID for each isotopologue of each fragment are denoted with an asterisk (*) and were established by considering the 95% confidence intervals of the measured CID. The complete dataset is available in Table S1.

Next, we evaluated the accuracy of CID measurements associated with the selected mass fragments by using ^13^C-PT standards. These standards harbored a binomial distribution for their carbon isotopologues for each fragment of each TMS derivative. For alanine_2TMS, both C2-C3 fragments showed a good accuracy, while C1 and C1-C2 fragments had some minor biases of measurements (Figure 2A). However, their statistical evaluation questioned the possibility of performing ^13^C-positional enrichment calculations for alanine. Regarding glutamate_3TMS, only the C2-C3-C4-C5_246 fragment (with M0 at *m*/*z* 246) had a good accuracy, while other fragments showed also minor biases of measurements with significant statistical differences (Figure 2B). Interestingly, all fragments for glycine_3TMS and serine_3TMS showed very good accuracies, except for the C1-C2-C3 fragment of serine_3TMS (Figure 2C,E). Nevertheless, it was still possible to calculate ^13^C enrichments at C1 and C3 positions by combining C2, C1-C2 and C2-C3 fragments. Mass fragments of proline_2TMS had the highest biases of measurements, except for the C2-C3-C4-C5_142 fragment (M0 at *m*/*z* 142) (Figure 2D). Finally, C2, C2-C3 and C4 mass fragments of malate_3TMS showed important biases of measurements compared to other fragments (Figure 2F). These results seriously questioned the ^13^C-positional enrichment calculations previously reported for C3 and C4 positions [29,34].

**Figure 3:**
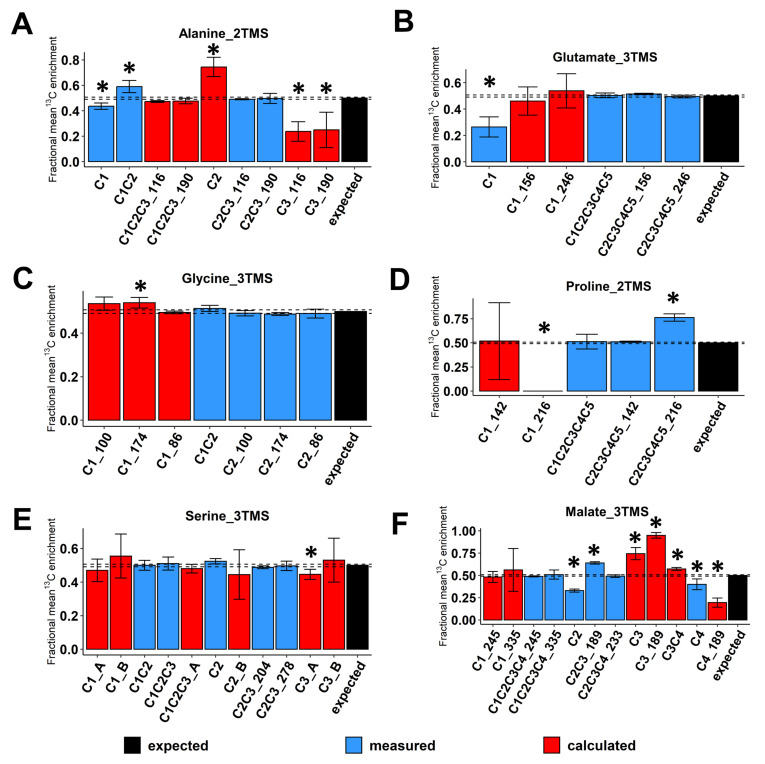
Evaluation of ^13^C-positional enrichment calculations for the selected mass fragments using ^13^C-PT standards. (**A**) Alanine_2TMS, (**B**) glutamate_3TMS, (**C**) glycine_3TMS, (**D**) serine_3TMS, (**E**) proline_2TMS, and (**F**) malate_3TMS. Black bars, expected mean ^13^C-enrichments; blue bars, measured mean ^13^C-enrichments; red bars, calculated mean ^13^C-enrichments. Values represent the mean ± SD for four biological replicates. Statistical differences between predicted and measured/calculated mean ^13^C enrichments for each isotopologue of each fragment are denoted with an asterisk (*) and were established by considering the 95% confidence intervals of the measured/calculated values. For convenience, fragments containing similar carbons were denoted by adding the *m*/*z* at M0 (see blue bars “C2_174”, “C2_100” and “C2_186” for glycine_3TMS, for example). When multiple fragments could be used to calculate the same ^13^C-positional enrichment, the original fragment used for calculation was denoted by adding its *m*/*z* at M0 (see red bars “C1_174”, “C1_100” and “C1_186” for glycine_3TMS, for example). The complete dataset in available in Table S1.

Then, we evaluated the accuracy of ^13^C-positional enrichment calculations (equations described in the material and methods). Indeed, ^13^C-PT standards had equal proportions of each possible isotopologue and each possible ^13^C-isotopomer leading to a positional mean ^13^C enrichment of 50% for each carbon of each metabolite [36]. For alanine_2TMS, we calculated ^13^C-enrichments for C1, C2 and C3 positions but also for the C1-C2-C3 molecule based on the C2-C3 fragment with M0 at either *m*/*z* 116 or 190 (Figure 3A). Overall, we showed that ^13^C-positional enrichment calculations for C2 and C3 positions were prone to large and statistically significant errors (calculated values around 0.75 and 0.20, respectively, compared to the expected value of 0.5). This result confirmed that the combination of small errors of measurements for C1 and C1-C2 fragments of alanine_2TMS resulted in larger errors after calculations. Thus, it is crucial to evaluate the measurement accuracy of mass fragments prior to application of ^13^C-positional GC-MS approaches to biological investigations. Besides this, the calculated fragment C1-C2-C3 for alanine_2TMS had a very good accuracy. For glutamate_3TMS, we only calculated ^13^C-enrichments for the C1 position based on the mass fragments available. While the direct measurement of ^13^C-enrichment for the C1 position was highly inaccurate (M0 at *m*/*z* 117), the combination of C1-C2-C3-C4-C5 and C2-C3-C4-C5_246 fragments surprisingly provided reasonable ^13^C-positonal enrichment values close to the expected 0.5 (Figure 3B). Thus, a combination of several fragments can represent a good alternative when some direct ions are inaccurate for MS measurements. For glycine_3TMS and serine_3TMS, the different combinations of accurate mass fragments yielded accurate ^13^C-positional enrichments for all calculations considered (Figure 3C,E). Nevertheless, for serine_3TMS, the combination “A” using C1-C2, C2-C3 and C2 fragments provided ^13^C-positional enrichments with smaller variability compared to combination “B” using C1-C2-C3, C1-C2 and C2-C3 fragments (see the C2 position, Figure 3E). For proline_2TMS, ^13^C-enrichment calculations at the C1 position using C1-C2-C3-C4-C5 and C2-C3-C4-C5_142 fragments were relatively accurate (mean value close to 0.5) but were subjected to an important variability (standard deviation close to ±0.4), thus preventing any further consideration for the study (Figure 3D). Finally, for malate_3TMS, the combination of C1-C2-C3-C4 fragments (M0 at *m*/*z* 245 or 335) with the C2-C3-C4 fragment allowed for the accurate calculation of ^13^C enrichment at the C1 position (Figure 3F). However, the fragment at *m*/*z* 245 showed a better resolution than the fragment at *m*/*z* 335, i.e., a small standard deviation after calculations. Conversely, the inaccuracy of mean ^13^C-enrichment measurements for C2, C2-C3 and C4 fragments led to statistically significant biases for calculations of ^13^C enrichments at C3 and C4 positions. Thus, only the C1 position was accurately determined for malate_3TMS. Overall, the results validated the use of some fragments and specific combinations to calculate accurate ^13^C-positional enrichments for the following atomic positions: (i) C1 and C2 of glycine_3TMS, (ii) C1, C2 and C3 of serine_3TMS, and (iii) C1 of malate_3TMS and glutamate_3TMS.

### 3.3. Application to Plant ^13^C-Labeled Experiments

Next, we used our validated ^13^C-positional approach to investigate key metabolic steps in plants by using incorporation kinetics of ^13^C-metabolic probes. For this purpose, we reanalyzed GC-MS spectra of a recent experiment, which followed the fate of U-^13^C-pyruvate into B. napus leaf discs during light or dark conditions [26]. Indeed, the light/dark regulation of photorespiration and PEPc activities introduced some differences of mean ^13^C-enrichments and isotopologues fractions into glycine, serine, malate and glutamate, but they were not investigated at the atomic position level.

First, we focused our analysis on mass fragments of glycine_3TMS and serine_3TMS. Previous results confirmed that decarboxylations from the TCA cycle were partly reassimilated by photosynthesis leading to ^13^C enrichments for glycine and serine under light conditions only (photorespiration).

**Figure 4:**
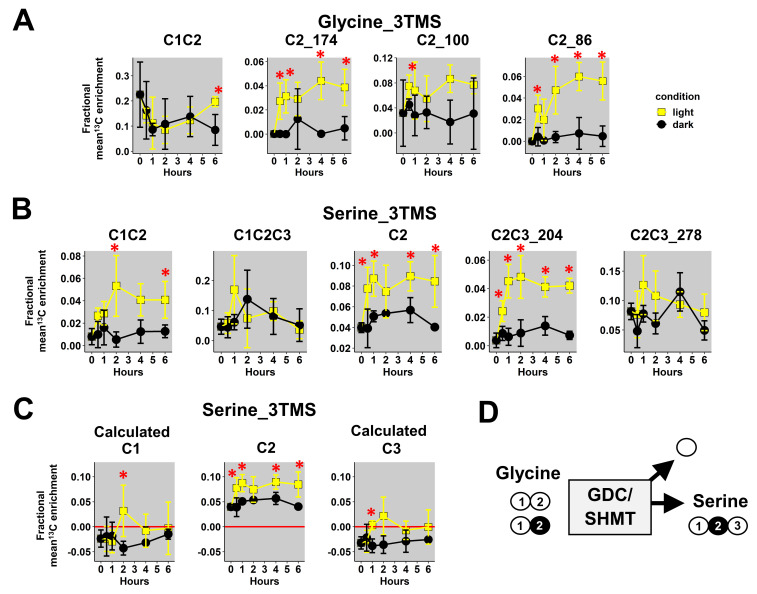
^13^C-positional enrichment analysis of glycine and serine during U-^13^C-pyruvate incorporation into B. napus leaf discs in light and dark conditions. (**A**) Glycine_3TMS, (**B**) serine_3TMS, (**C**) calculated positions for serine_3TMS, and (**D**) metabolic explanation. Black points, dark conditions; yellow points, light conditions. Values represent the mean ± SD for four biological replicates. Statistical differences between light and dark conditions are denoted with red stars according to a Wilcoxon–Mann–Whitney test (*, *p*-value < 0.05). The complete dataset is available in Table S2.

Here, we observed a statistically significant increase of mean ^13^Cenrichments for the three fragments containing the C2 (M0 at *m*/*z* 174, 100 and 86) in light conditions compared to dark conditions (Figure 4A). There were no major differences for the C1-C2 fragment in both conditions, but it had a large biological variability in plant samples (mean value of 0.2 ± 0.1). Therefore, it was not possible to use a ^13^C-positional approach for glycine_3TMS. However, C1-C2, C2 and C2-C3_204 fragments of serine_3TMS showed statistically significant increases in ^13^C enrichment in light conditions compared to dark conditions with a relatively small biological variability (Figure 4B). Using the combination “A”, we calculated the ^13^C enrichment for C1 and C3 positions. Overall, the calculated ^13^C positional enrichments were below 0, and there were no major statistical differences between light and dark conditions except for the C2 position (Figure 4C). Thus, our ^13^C-positional approach formerly showed that serine was only labeled at the C2 position in light conditions during the experiment. Based on the properties of the glycine decarboxylase (GDC) and serine hydroxymethyl aminotransferase (SHMT), serine was produced from the combination of non-labeled glycine and ^13^C-2-glycine (^13^C at C2 position) (Figure 4D). Thus, we could also deduce that the C1 position of glycine_2TMS was not ^13^C labeled during the experiment.

**Figure 5:**
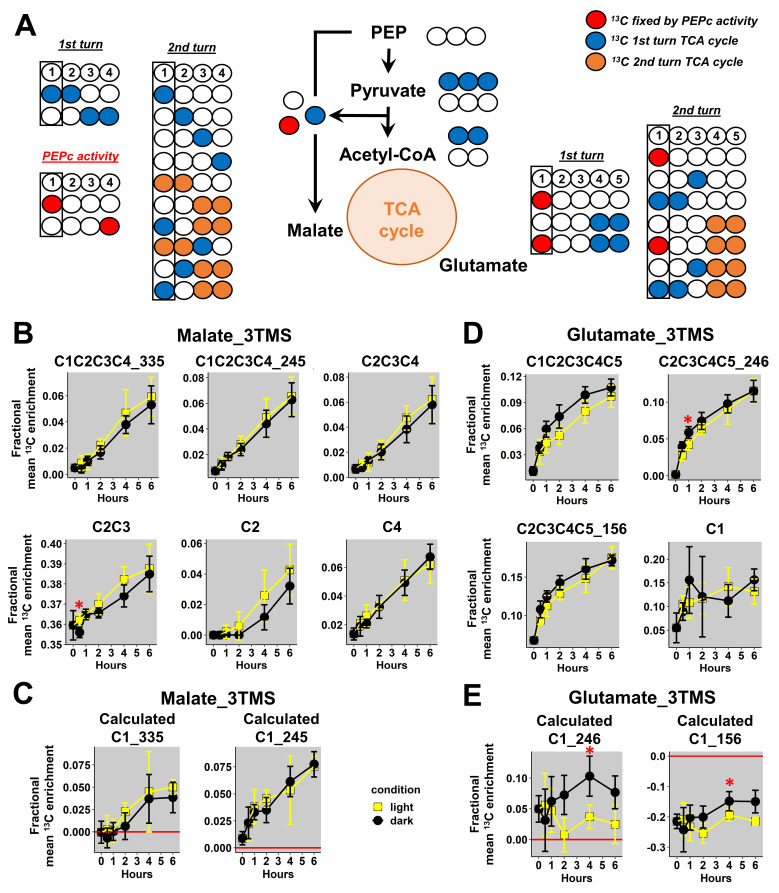
^13^C-positional enrichment analysis of malate and glutamate during U-^13^C-pyruvate incorporation into B. napus leaf discs in light and dark conditions. (**A**) Metabolic network and possible isotopologues, (**B**) malate_3TMS, (**C**) calculated positions for malate_3TMS, (**D**) glutamate_3TMS, and (**E**) calculated positions for glutamate_3TMS. Black points, dark conditions; yellow points, light conditions. Values represent the mean ± SD for four biological replicates. Statistical differences between light and dark conditions are denoted with red stars according to a Wilcoxon–Mann–Whitney test (*, *p*-value < 0.05). The complete dataset is available in Table S2.

Then, we focused our analysis on mass fragments of malate_3TMS and glutamate_3TMS. Previous analysis of the considered metabolic network showed that the C1 position of malate_3TMS and glutamate_3TMS could be ^13^C labeled through the activities of PEPc and TCA cycle enzymes (Figure 5A). Thus, the evaluation of ^13^C enrichment at the C1 position for these metabolites reflected the light-dependent regulation of both pathways [44,45]. Here, the additional mass fragments analyzed for malate_3TMS had good resolutions (small standard deviations), but there were no formal differences between light and dark conditions (Figure 5B). However, the C2-C3 fragment had a strong bias at natural ^13^C abundance, with a mean ^13^C enrichment starting at 0.36 at the “0” time point. Therefore, this fragment could not be considered for any isotopic study. Overall, the calculated ^13^C enrichment for the C1 position was relatively similar when using the two fragments (*m*/*z* 335 and 245) and was not significantly different between light and dark conditions (Figure 5C). Thus, the decrease of TCA cycle activity in light may have been compensated by an increase of PEPc activity leading to a similar ^13^C enrichment at the C1 position in both conditions. Regarding glutamate_3TMS, we also obtained good resolutions (small standard deviations) for the additional mass fragments analyzed, except for the C1 fragment, which may reflect its poor accuracy previously identified with ^13^C-PT standards (Figure 5D). Nevertheless, measurements at natural ^13^C abundance for the C2-C3-C4-C5_156 fragment was slightly biased in plant samples (around 0.06 at the “0” time point) and could explain the large bias identified after calculations (Figure 5E). Besides this, there were no statisticaldifferences between light and dark conditions for the mass fragments, although a trend could be observed in the C1-C2-C3-C4-C5 fragment (higher values in dark conditions than in light conditions) (Figure 5D). Calculations of the ^13^C-positional enrichment for the C1 position of glutamate_3TMS were only validated when using the C2-C3-C4-C5_246 fragment in plant samples (Figure 5E). Interestingly, this calculation successfully identified a statistically significant increase in ^13^C enrichment at the C1 position in dark conditions compared to light conditions after 4 h of labeling. However, this ^13^C-positional enrichment was not formerly modified during light conditions (along the kinetic), hence questioning the importance of PEPc and the cyclic mode of the TCA cycle in contributing to glutamate biosynthesis in the light. Therefore, this interesting result may essentially reflect a higher contribution of the cyclic mode of the TCA cycle to glutamate biosynthesis in the dark.

## 4. Discussion

### 4.1. Identification of Mass Fragment-Specific Analytical Biases and Consequences for GC/MS-Based ^13^C-Positional Approaches

In this study, we evaluated the accuracy of additional mass fragments for some TMS derivatives using ^13^C-PT standards and their use for ^13^C-positonal approaches. Overall, we showed that some mass fragments of proline_2TMS, glutamate_3TMS, malate_3TMS and α-alanine_2TMS had important biases for the measurement of mean ^13^C enrichments.

For proline_2TMS, we observed a large and significant inaccuracy for CID measurements with the C2-C3-C4-C5 fragment (M0 at *m*/*z* 216). Analysis of MS spectra confirmed the presence of a contaminant ion at *m*/*z* 218 in both standard mixtures and plant samples visible at a natural ^13^C abundance (Figure S1A). Interestingly, the C2-C3-C4-C5 fragment with M0 at *m*/*z* 142 was already reported as weakly inaccurate due to a contamination at *m*/*z* 146 by the co-elution of isoleucine [26,46]. Since the EI-induced fragmentation of isoleucine_2TMS also produced a massive ion at *m*/*z* 218, the contamination was due to co-elution issues. Nevertheless, the fragment at *m*/*z* 142 accurately calculated ^13^C enrichment at the C1 position for proline_2TMS (Figure 3B). Thus, this last fragment should be preferentially selected for ^13^C-labeling studies compared to the fragment at *m*/*z* 216.

Regarding the recent establishment of a GC/MS-based ^13^C-positional approach, we identified analytical biases for some fragments of glutamate and malate leading to large errors for calculations of ^13^C-positional enrichments. Notably, we showed that the C1 fragment of glutamate_2TMS largely underestimated the level of ^13^C-enrichment with a value of 0.25 compared to the theoretical value of 0.5 (Figure 3D). A previous analysis by high resolution mass spectrometry confirmed the presence of a contaminant mass at *m*/*z* 117 but used a linear correlation between calculated and measured ^13^C enrichments at the C1 position to correct for this bias [29]. However, this correction assumed a linear propagation of the bias and did not consider other possible biases for ^13^C-positional enrichment calculations. Here, we confirmed that both C2-C3-C4-C5 fragments (M0 at *m*/*z* 156 and 246) can be combined with the C1-C2-C3-C4-C5 fragment to calculate accurate ^13^C enrichments at the C1 position. However, we observed a factor of two between calculated and measured ^13^C-positional enrichments compared to the previous study, which found a factor of five. Thus, the bias associated to the C1 fragment at *m*/*z* 117 may not follow a linear propagation. Therefore, ^13^C enrichment at position C1 of glutamate_2TMS should always be calculated instead of directly measured with the fragment at *m*/*z* 117.

Besides this, we showed that the C2 (*m*/*z* 265), C2-C3 (*m*/*z* 189) and C4 (*m*/*z* 117) fragments of malate_3TMS contained significant biases for measurements of ^13^C enrichments leading to large errors in ^13^C-positional enrichment calculations at C3 and C4 positions (Figure 2F and Figure 3F). For the C2-C3 fragment of malate_3TMS, an exact mass GC-MS analysis identified a similar mass fragment at the same retention time arising from threitol_4TMS (*m*/*z* 189.07 versus *m*/*z* 189.11) [47]. Thus, a contamination by this compound in ^13^C-PT standards and plant samples was potentially expected. However, both reference chemical standards (^12^C) and plant samples had the same magnitude of inaccuracy for measurements of mean ^13^C enrichment and CIDs at natural ^13^C abundance (Figure S1). Thus, an analytical bias during the fragmentation process seems a more plausible explanation. For the C4 fragment of malate_3TMS, its chemical structure was unequivocally identified from injections of ^13^C standards labeled at specific positions [31]. A recent analysis by high resolution mass spectrometry revealed the presence of a contaminant mass at a weak intensity level (34% of *m*/*z* 117.0731 versus 66% of *m*/*z* 117.0366 (C4 fragment)) [29]. The authors proposed a chemical structure containing the C2 and C3 carbons based on mass fragmentation simulations and injections and a basic correction step based on the relative abundance of these two ions. However, this correction step did not consider: (i) the combination of multiple isotopic clusters starting at the same M0 for the correction of naturally occurring isotopes and (ii) possible biases arising from ^13^C-labeled C2 and C3 positions. In this study, we measured an important bias, i.e., a mean ^13^C enrichment of 0.4 compared to the expected value of 0.5. Thus, the use of the C4 fragment should only be considered for a qualitative evaluation of PEPc activity rather than an accurate measurement suitable for ^13^C-metabolic flux analysis.

Overall, our analysis revealed that measurements and calculations of ^13^C-positional enrichments for malate_3TMS were largely inaccurate except at the C1 position. Since some biases arise from mass contaminations, the use of high-resolution mass spectrometry will help to improve the accuracy of the GC/MS-based ^13^C-positional approach [47]. The use of TBDMS derivatives instead of TMS derivatives could also constitute an interesting alternative. A GC-tandem MS fragmentation approach was applied to TBDMS derivatives and succeeded at measuring the complete isotopomer distribution of aspartate [30]. This method relied on redundant measurements of carbon atom enrichments from different combinations of precursor and product ions followed by estimations of isotopomer distribution with least-squared regressions. Interestingly, aspartate represents a good proxy to evaluate the ^13^C composition of TCA cycle intermediates (oxaloacetate and malate) [30]. Since TBDMS derivatives of amino acids are more stable than TMS derivatives [26,48], this strategy seems highly promising for the field of plant science. In addition, a fragmentation map of precursor and product ions has been recently established for TBDMS derivatives of citrate and malate [31].

### 4.2. A GC/MS-Based ^13^C-Positional Approach Suitable for Investigating the Metabolic Fluxes Associated to Photorespiration, TCA Cycle and PEPc

Using ^13^C-PT standards, we validated a GC/MS-based ^13^C-positional approach for the following atomic positions: (i) C1 and C2 of glycine_3TMS, (ii) C1, C2 and C3 of serine_3TMS, and (iii) C1 of malate_3TMS and glutamate_3TMS. We showed that this approach could be successfully applied to plant ^13^C-labeled experiments for investigating key metabolic fluxes of plant primary metabolism. Photorespiration is a major flux involved in the recycling of by-products from RuBiSCO oxygenase activity [49,50]. Analysis of Arabidopsis mutant lines confirmed that the GDC/SHMT enzymes catalyzed the rate-limiting step of the photorespiratory flux by converting two molecules of glycine into one molecule of serine, CO_2_ and NH_3_ [51]. Since photorespiration is associated to carbon losses, crop improvement can require the accurate measurement of this metabolic flux with a ^13^C-labeling strategy [52]. Previous analysis showed that NMR was best suited for obtaining positional information into serine compared to LC-MS [53]. Here, we showed that GC-EI-MS can be highly accurate by combining multiple mass fragments. Since NMR analysis requires large amounts of material compared to MS analysis, this methodological improvement certainly represents a decisive advantage for ^13^C-metabolic flux analysis.

Regarding TCA cycle and its PEPc anaplerotic pathway, the positional approach provided additional information compared to previous results obtained only with isotopologues of malate and glutamate in B. napus. Indeed, a ^13^C-metabolic flux analysis with pathway-specific isotopologues showed that PEPc-dependent reassimilation of TCA cycle-derived CO_2_ had a small contribution to citrate biosynthesis in the light compared to TCA cycle [26]. These specific isotopologues included M1, M2 and M3 from glutamate_2TMS_C2C5 and malate_2TMS_C2C4 fragments and suggested the occurrence of a cyclic mode of the TCA cycle in the light. Here, we did not identify an increase of ^13^C enrichment at the C1 position for glutamate in the light conditions. Thus, the isotopologues produced by the TCA cycle in the light should not contain this labeling pattern (Figure 5A). However, such a scenario is practically impossible because (i) PEPc activity fixes ^13^C labeling at the C4 position of malate first, prior to a reversible fumarase step producing the same ^13^C-labeling at the C1 position of malate; and (ii) the contribution of PEPc to citrate biosynthesis has been formerly identified in the same experiment. Besides this, a recent analysis of U-^13^C-pyruvate incorporation in Arabidopsis leaves in the light provided contrasted results for ^13^C enrichment at the C1 position of glutamate [29]. The authors identified an increase in ^13^C enrichment for this C1 position and for the fragment *m*/*z* 246 containing the C2-C3-C4-C5 positions. However, these authors used the *m*/*z* 117 of glutamate_3TMS for the C1 positional analysis, i.e., a fragment that is prone to large biases (Figure 2B and Figure 3B). Therefore, the lack of ^13^C enrichment at the C1 position of glutamate in our experiment (light conditions) could be due to a weak accuracy at small ^13^C enrichments. Indeed, we observed significant differences for ^13^C enrichment at the C1 position of glutamate_2TMS between light and dark conditions that had an obvious biological explanation. The TCA cycle has a higher turnover in dark conditions compared to light conditions [45], and a second turn of the TCA cycle can produce ^13^C labeling at the C1 position of glutamate [26]. Thus, this positional approach seems to be well suited for investigating metabolic fluxes but only to compare light and dark conditions. Besides this, our analysis of ^13^C enrichment at the C1 position of malate did not reveal differences between light and dark conditions in B. napus leaves (Figure 5B,C). Since PEPc activity is inhibited in the dark compared to light conditions, our results suggested that the decrease in PEPc activity in the dark was perhaps compensated by higher TCA cycle activity. In this situation, a similar ^13^C enrichment at the C1 position of malate was expected for both light and dark conditions.

## 5. Conclusions

The accuracy of GC/MS-based ^13^C-positional approaches must be evaluated prior to further deployment for scientific investigations. For this purpose, tailor-made ^13^C-PT standards are a valuable resource for evaluating biases in CID measurements and in ^13^C-positional enrichment calculations. Overall, we showed that some mass fragments of proline_2TMS, glutamate_3TMS, malate_3TMS and α-alanine_2TMS had important biases for measurements of mean ^13^C enrichments. These biases were further transmitted to ^13^C-positional enrichments during calculations leading to larger biases. Nevertheless, we validated a GC/MS-based ^13^C-positional approach for the following atomic positions: (i) C1 and C2 of glycine_3TMS, (ii) C1, C2 and C3 of serine_3TMS, and (iii) C1 of malate_3TMS and glutamate_3TMS. We successfully applied this approach to plant ^13^C-labeling experiments and obtained complementary information regarding the light/dark regulation of the photorespiratory production of serine and the biosynthesis of malate and glutamate through the TCA cycle and PEPc activities.

## Figures and Tables

**Table 1:** Detailed list of the fragments considered in this study. TMS, C_3_H_9_Si.

TMS Derivative	Loss		Fragment		
	Formula	Mass	Formula	*m*/*z*	Carbon Backbone
Alanine(2TMS)	CH_3_	15	C_8_H_20_NO_2_Si_2_	218	1-2
	CH_3_, CO	43	C_7_H_20_NOSi_2_	190	2-3
	TMS-CO_2_	117	C_5_H_14_NSi	116	2-3
	unknown	130	C_4_H_11_OSi	103	1
Glutamate(3TMS)	CH_3_	15	C_13_H_30_NO_4_Si_3_	348	1-2-3-4-5
	TMS-CO_2_	117	C_10_H_24_NO_2_Si_2_	245	2-3-4-5
	TMS-CO_2_, TMS-OH	207	C_7_H_14_NOSi	156	2-3-4-5
	TMS-NH-C_3_H_5_-CO_2_-TMS	246	C_4_H_9_O_2_Si	117	1
Glycine(3TMS)	CH_3_	15	C_10_H_26_NO_2_Si_3_	276	1-2
	CH_3_, CO	43	C_9_H_26_NOSi_3_	248	2
	TMS-CO_2_, TMS-H	191	C_4_H_10_NSi	100	2
	TMS-CO_2_, TMS-CH_3_	205	C_3_H_8_NSi	86	2
Malate(3TMS)	CH_3_	15	C_12_H_27_O_5_Si_3_	335	1-2-3-4
	C_3_HO_3_	85	C_10_H_29_O_2_Si_3_	265	2
	TMS-OH, CH_3_	90	C_9_H_17_O_4_Si_2_	245	1-2-3-4
	TMS-CO_2_	117	C_9_H_21_O_3_Si_2_	233	2-3-4
	TMS-CO_2_, CH_3_, COH	161	C_7_H_17_O_2_Si_2_	189	2-3
	C_9_H_21_O_3_Si_2_	233	TMS-CO_2_	117	4
Proline(2TMS)	CH_3_	15	C_10_H_22_NO_2_Si	244	1-2-3-4-5
	CH_3_, CO	43	C_9_H_22_NOSi	216	2-3-4-5
	TMS-CO_2_	117	C_7_H_16_N	142	2-3-4-5
Serine(3TMS)	CH_3_	15	C_11_H_28_NO_3_Si_3_	306	1-2-3
	CH_3_, CO	43	C_10_H_28_NO_2_Si_3_	278	2-3
	TMS-CH_2_O	103	C_8_H_20_NO_2_Si_2_	218	1-2
	TMS-CO_2_	117	C_8_H_22_NOSi_2_	204	2-3
	unknown	221	C_4_H_10_NSi	100	2

## Data Availability

The data presented in this study are available in article and Supplementary Material.

## Supplementary Materials

The following supporting information can be downloaded at: https://www.mdpi.com/article/10.3390/metabo13040466/s1. Figure S1, Analytical biases identified for the C2C3C4C5_216 fragment of proline_2TMS and the C2C3_189 fragment of malate_3TMS; Table S1, Complete dataset for 13C-PT standards; Table S2, Complete dataset for U-13C-pyruvate incorporation experiments.
