# Supplementary material for: Evaluation of GC/MS-Based 13C-Positional Approaches for TMS Derivatives of Organic and Amino Acids and Application to Plant 13C-Labeled Experiments
Source: Metabolites. 2023 Mar 23;13(4):466. doi: 10.3390/metabo13040466 (PMC10142191; doi:10.3390/metabo13040466)
Supplement: Supplementary file 1 [file metabolites-13-00466-s001.zip › Figure S1.pdf]

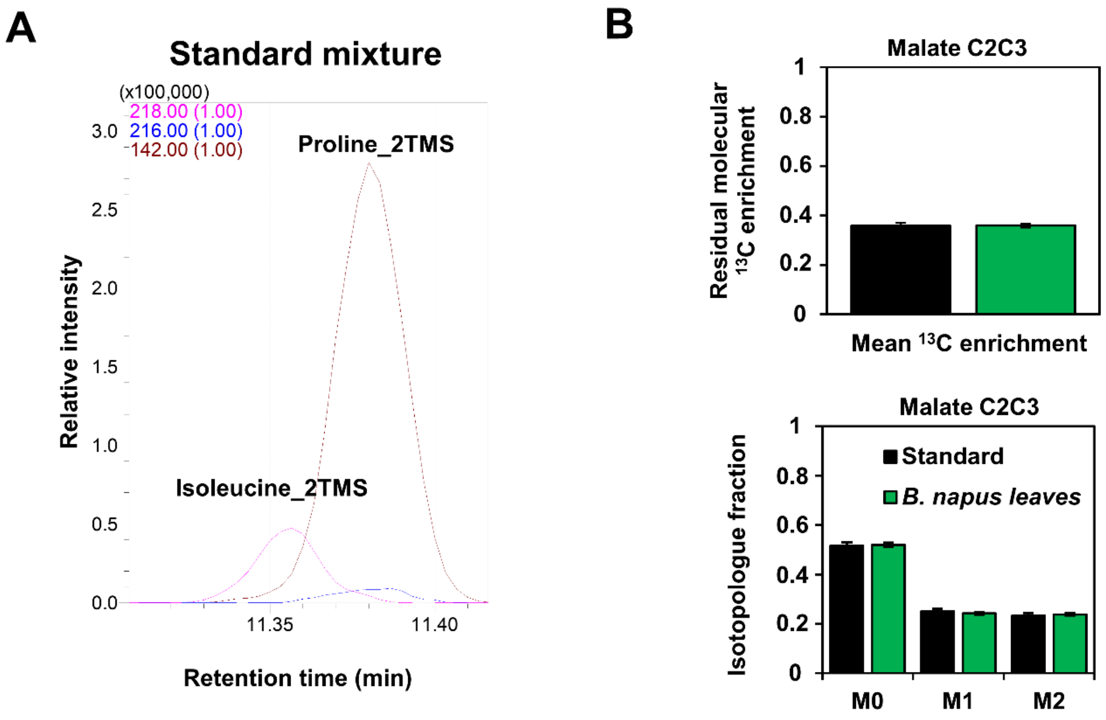

Figure S1. Analytical biases identified for the C2C3C4C5_216 fragment of proline_2TMS (**A**) and the C2C3_189 fragment of malate_3TMS (**B**, **C**).
